# Gene expression in early and progression phases of autosomal dominant polycystic kidney disease

**DOI:** 10.1186/1756-0500-1-131

**Published:** 2008-12-21

**Authors:** Wen-Cheng Chen, Yi-Shiuan Tzeng, Hung Li

**Affiliations:** 1 Institute of Biochemistry and Molecular Biology, National Yang-Ming University, 155 Linong Street, Taipei 112, Taiwan; 2 Institute of Molecular Biology, Academia Sinica, 128 Academia Road, Taipei 115, Taiwan

## Abstract

**Background:**

Little is known about the genes involved in the initial cyst formation and disease progression in autosomal dominant polycystic kidney disease (ADPKD); however, such knowledge is necessary to explore therapeutic avenues for this common inherited kidney disease.

**Findings:**

To uncover the genetic determinants and molecular mechanisms of ADPKD, we analyzed 4-point time-series DNA microarrays from *Pkd1*^*L*3/*L*3 ^mice to generate high resolution gene expression profiles at different stages of disease progression. We found different characteristic gene expression signatures in the kidneys of *Pkd1*^*L*3/*L*3 ^mice compared to age-matched controls during the initial phase of the disease. By postnatal week 1, the *Pkd1*^*L*3/*L*3 ^kidney already had a distinctive gene expression pattern different from the corresponding normal controls.

**Conclusion:**

The genes differentially expressed, either induced or repressed, in ADPKD are important in immune defense, cell structure and motility, cellular proliferation, apoptosis and metabolic processes, and include members of three pathways (Wnt, Notch, and BMP) involved in morphogenetic signaling. Further analysis of the gene expression profiles from the early stage of cystogenesis to end stage disease identified a possible gene network involved in the pathogenesis of ADPKD.

## Findings

ADPKD is one of the most common lethal genetic diseases in the world, with a prevalence of about one in every 400 to 1000 persons and a 50% chance that an ADPKD patient will die of kidney failure [1-3]. Early changes in the disease process are very important, and unraveling more of the initial steps in pathogenesis may afford patients better management possibilities and an improved prognosis. Determining the molecular mechanism of the pathogenesis of ADPKD, including the early period of cyst formation, requires investigation of changes from before the earliest period of disease manifestation in a defined animal model that mimics the human disease [4-6]. One well-used approach to unravel the molecular pathogenesis and pharmacology of metabolic and genetic diseases is the cDNA microarray [7-9]. We have previously developed an animal model of ADPKD by generating *Pkd1*^*L*3/*L*3 ^mutant mice [10]. Homozygous *Pkd1 *null mice appear normal when born, but rapidly develop polycystic kidneys and generally do not live longer than 3.5 to 4 weeks. Here we refined the genetic background of our *Pkd1*^*L*3/*L*3 ^mutant mice so that the disease more closely resembles most human ADPKD individuals in the late onset of symptoms and final progression to end-stage renal disease (ESRD). This refined animal model will facilitate the study of ADPKD progression and the evaluation of possible treatments. In this paper we present the gene expression profile of *Pkd1*^*L*3/*L*3 ^mice and their normal littermates at different time points, as determined by cDNA microarray. The materials and methods used in this study were described detail in the additional file 1.

## Results

### Characterization of *Pkd1*^*L*3/*L*3 ^Mice on a Congenic C57BL/6 Background

During the course of refinement of the genetic background of the ADPKD model mice to a congenic C57BL/6 genetic background, an earlier onset of polycystic kidney phenotype (see Additional file 2) was seen than with the previous mice on the SV129/C57BL6 background. Histological examination revealed that the translucent enlarged kidneys were due to the formation of numerous large cysts in homozygous mutant mice. Smaller cysts formed early in PNW 1, and became larger at PNW 2. The cysts were disseminated and distributed cross the cortex and medulla. At PNW 3.5, the cyst occupied the entire kidney and normal kidney architecture was hardly seen (see Additional file 2C). All *Pkd1*^*L*3/*L*3 ^mice were born normally, but most of them did not survive past four weeks (see Additional file 3A) in the congenic C57BL/6 background. In gross appearance, there was no difference between *Pkd1*^*L*3/*L*3 ^mice and their age-matched control littermates at PNW 1, but *Pkd1*^*L*3/*L*3 ^mice were shorter of stature with a slightly wider abdomen by PNW 2, and more obviously so by PNW 3 (see Additional file 2A). On necropsy, these homozygous mutant mice had much enlarged and translucent kidneys compared to their heterozygous or wild-type littermates (see Additional file 2B). The gross kidney alterations were first seen at PNW 2 and became more severe at later time points. Although the body weight was only moderately reduced in homozygous mutants (see Additional file 3B), there was a large increase in kidney weight/body weight ratio (kw/bw) (see Additional file 3C) and kidney volume (see Additional file 3D) in homozygous mutant mice. Similar changes in kidney volume were seen in *Pkd1*^*L*3/*L*3 ^mice compared to the control littermates. Renal function was also severely impaired in homozygous mutant mice as evidenced in the progressive rise in BUN (see Additional file 3E).

### Overview of Temporal Expression Profile of *Pkd1*^*L*3/*L*3 ^Kidney

To study the detailed gene expression profile of ADPKD, we generated and compared gene expression profiles of *Pkd1*^*L*3/*L*3 ^mice and their aged-matched wild-type littermates at different stages, i.e. PNW 1, 2, 3 and 3.5.

**Figure 1:**
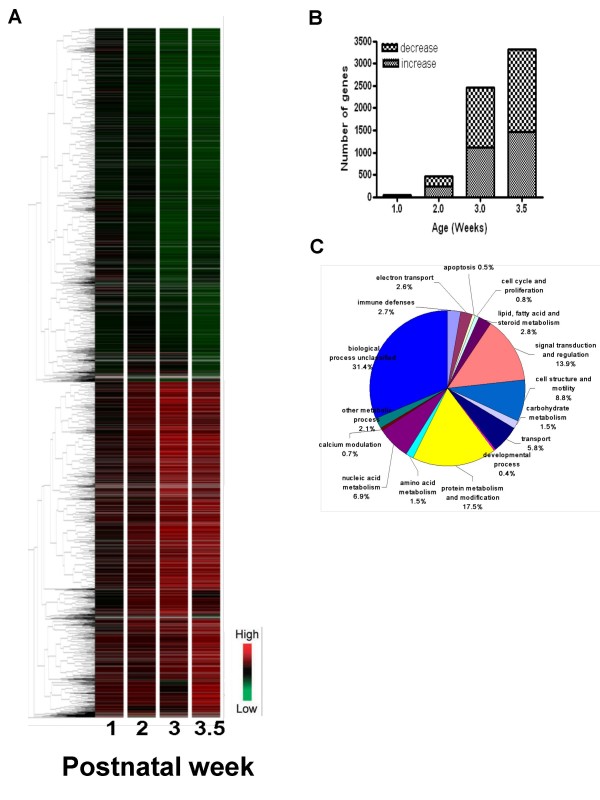
**Relative gene expression levels in *Pkd1*^*L*3/*L*3 ^kidney compared to control**. (A) Hierarchical clustering of *Pkd1*^*L*3/*L*3^/wild-type expression ratios for 4,231 genes differentially expressed in kidney at one or more ages between PNW 1 and PNW 3.5 (replicate samples normalized to wild-type at each age). Genes more highly expressed in *Pkd1*^*L*3/*L*3 ^mice are indicated in red; those expressed in *Pkd1*^*L*3/*L*3 ^mice at levels lower than wild-type mice are indicated in green. Red and green represent up- and downregulation, respectively (see scale in lower right corner). Ten gene clusters were identified as labeled at right. Vertical black bars on the sides (1,917 downregulated genes in A1–A5, and 1,806 upregulated genes in A6–A10) indicate regions with characteristic gene expression change patterns. (B) Numbers of differentially regulated genes by age in *Pkd1*^*L*3/*L*3 ^mice. (C) Functional class proportions of all genes differentially expressed in *Pkd1*^*L*3/*L*3 ^and wild-type mice from all time points (PNW 1, 2, 3 and 3.5).

There were significant differences in expression profile seen at all timepoints examined (Figure 1A). 4,231 genes were found to be differentially expressed in the *Pkd1*^*L*3/*L*3 ^kidney at one or more timepoint (Figure 1B and see Additional file 4), and these genes were assigned to one of 16 functional categories (Figure 1C) based on a previous classification. The number of genes in each functional class that differed at each time point of the disease was then determined (see Additional file 5) and the top 10 genes in each group according to their gene expression ratio at PNW 3.5 were listed (see Additional file 6).

### Validation of Genes Involved in the Wnt, Notch, and BMP Signaling Pathway

**Figure 2:**
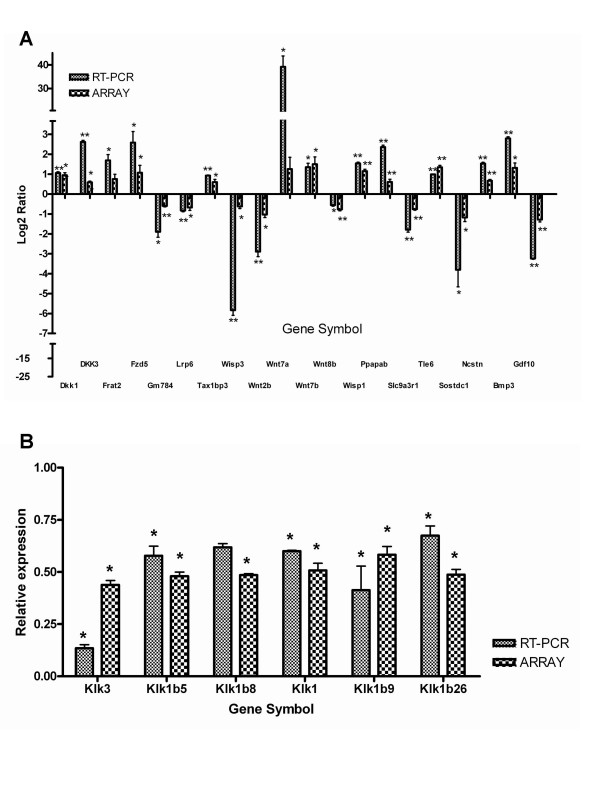
**Validation of genes involved in the Wnt, Notch and BMP signaling pathways and early differentially expressed genes at PNW 1**. (A) Twenty differentially expressed genes from the microarray were validated by RT-PCR using *Gapdh *as a reference gene. The time point that showed the greatest relative change in the microarray was selected for RT-PCR. (B) RNA was extracted from *Pkd1*^*L*3/*L*3 ^mice and age-matched control littermates at PNW 1. The RT-PCR and microarray results are compared in the same figure (mean ± SD of triplicate PCR reactions of pooled RNA; *n *= 3; *, *P *< 0.05; **, *P *< 0.01 compared with wild-type mice, by Student's *t*-test).

In addition, several genes differentially expressed in this model are components of three morphogenetic signaling pathways. Quantitative real time PCR was performed to validate gene expression changes identified by microarray analysis during disease progression (see Additional file 7 for details of primers and conditions). Genes involved in the Wnt, Notch and BMP signaling pathway were examined, and most gene expression patterns were consistent with the microarray results (Figure 2A).

### Transcriptome Alteration in *Pkd1*^*L*3/*L*3 ^Kidney in the Early Phase of ADPKD

An original goal of this study was to identify gene expression alterations in *Pkd1*^*L*3/*L*3 ^kidney during the initial and progressive phases, which may represent specific landmarks in a molecular signature of ADPKD. A total of 50 genes met these criteria by PNW 1 (see Additional file 8): 16 upregulated genes and 34 downregulated genes. The genes affected were virtually all implicated in adaptive and/or pathogenic mechanisms that might be linked to cystogenesis or disease progression (see Additional file 8). To further confirm the differential gene expression at PNW1 observed with the PKD custom microarray and suggested by IPA as being important, we validated the differential expression of the *Klk *gene family using quantitative real-time PCR analysis normalized against the housekeeping gene *Gapdh*. The genes selected were *Klk3, Klk1b5, Klk1b8, Klk1b26, Klk1 *and *Klk1b9*. The expression pattern of all selected genes was consistent with the microarray results (Figure 2B). In general, PCR-based estimates of expression change were larger than those observed in the microarray study. These genes may prove useful as potential therapeutic target candidates.

### Pathway Analysis of Differentially Expressed Genes in *Pkd1*^*L*3/*L*3 ^Mice at Initial and Progressive Phase

To gain insight into the potential functional consequences of differentially expressed genes in *Pkd1*^*L*3/*L*3 ^mice, the differentially expressed genes were analyzed by the program IPA (Table 1). IPA identified 4 significant networks among the early genes altered in the *Pkd1*^*L*3/*L*3 ^mice at PNW 1, associated with cardiovascular system development and function, gene expression, and cell morphology (Table 1). We also analyzed PNW 2, 3 and 3.5 data with IPA (see Additional file 9), and we found other network might be involved in the pathogenesis of ADPKD during disease progression, such as tissue morphology, development, and cellular movement.

**Table 1:** Networks generated from IPA for differentially expressed genes in the Pkd1^L3/L3 ^mice at PNW 1^a^

Network ID	Genes in network	Score	# of Focus Genes	Top categories
1	**↓ABCG2**, **↑ALDH1A1**, **↓CTSL2**, **↓F13B**, **↓GATM**, **↓IDH1**, **↑IL17RB**, **↓KLK3**, **↓KLK1 **(includes EG:16612), **↓KLK1**, **↓KLK1B9**, **↓KLK1B26**, **↓PAH**, **↑VWF**	36	14	Cardiovascular System Development and Function, Gene Expression, Cell Morphology
2	**↓ACSS1**, **↓EGFR**, **↑ KRT18**, **↓SLC2A2**	7	4	Gene Expression, Cell Cycle, Cell Death
3	**↑GLDN**	3	1	Cellular Movement, Nervous System Development and Function, Cancer
4	**↑AADAC**	3	1	Carbohydrate Metabolism, Cancer, Hepatic System Disease

^*a*^Genes whose expression was either increased or decreased in *Pkd1*^*L*3/*L*3 ^mice as a function of age were identified by correlation analysis across four time points: 1,2, 3 and 3.5 weeks.

### Correlation Analysis Identifies Genes Progressively Induced or Repressed with Disease

**Figure 3:**
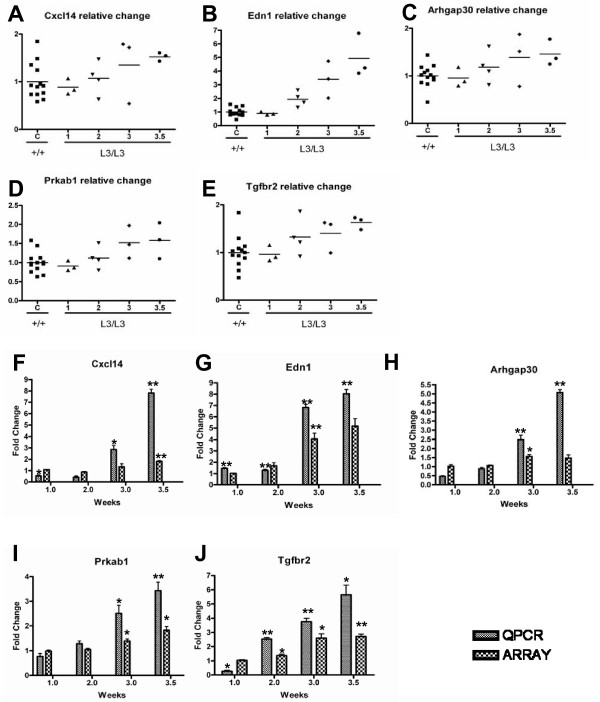
**Selected genes which might be related to disease progression from correlation analysis and RT-PCR validation**. Genes whose expression increased or decreased with disease progression were identified by correlation analysis of microarray data (A~E). Changes in transcript abundance are expressed as amount of change relative to control mean for that gene, and individual values of three different mice and the mean are shown at each week. (F~J) Validation of the microarray results by QRT-PCR analysis, showing relative change of PCR signal between wild-type and *Pkd1*^*L*3/*L*3 ^mice (mean ± SD of triplicate PCR reactions of pooled RNA; *n *= 3; *, *P *< 0.05; **, *P *< 0.01 compared with wild-type mice, by Student's *t*-test).

In order to find genes related to disease progression, the Pearsons' correlation coefficient of affected genes of *Pkd1*^*L*3/*L*3 ^mice at PNW 1, 2, 3 and 3.5 was calculated. A small number of genes whose expression was constant in control mice but whose expression either continuously increased or decreased in *Pkd1*^*L*3/*L*3 ^mice was identified, and the 5 annotated genes most well correlated to disease progression (with *P *values lower than 0.05) are shown in Table 2 and Figure 3A–E. RT-PCR was performed to validate these five genes. The expression of these genes increased progressively, consistent with the microarray results (Figure 3F–J).

**Table 2:** Top-scoring genes from correlation analysis^a^

Gene symbol	Description	Correlation	***P***-value
*Cxcl14*	Chemokine ligand 14	0.999	1.14E-03
*Edn1*	Endothelin 1	0.998	2.25E-03
*Arhgap30*	Rho GTPase activating protein 30	0.992	7.85E-03
*Prkab1*	Protein kinase AMP-activated beta 1 non-catalytic subunit	0.990	9.61E-03
*Tgfbr2*	Transforming growth factor, beta receptor II	0.959	4.07E-02

^*a*^Genes whose expression was either increased or decreased in *Pkd1*^*L*3/*L*3 ^mice as a function of age were identified by correlation analysis across four time points: 1,2, 3 and 3.5 weeks.

### IHC Staining of Transforming Growth Factor Receptor (Tgfβr) 1 and 2 Expression

**Figure 4:**
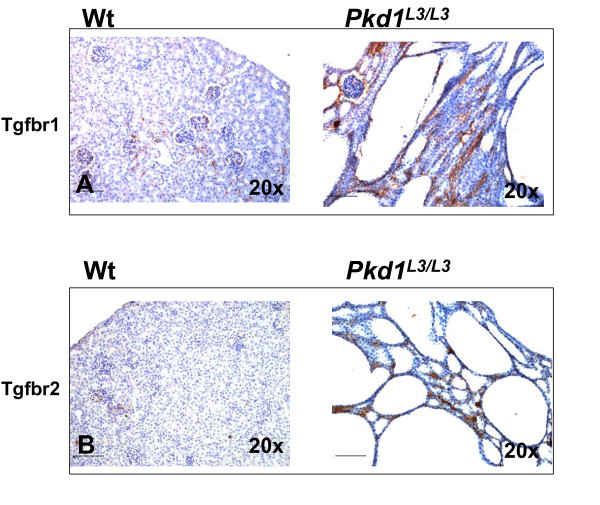
**Elevated expression of TGFβR1 and TGFβR2 in *Pkd1*^*L*3/*L*3 ^and control mice at PNW 3.5**. Immunohistochemistry of kidney of *Pkd1*^*L*3/*L*3 ^and control mice at PNW 3.5, using monoclonal antibody to TGF-β receptor subtypes 1 and 2, visualized with diaminobenzidine (DAB). Representative images for Tgfbr1 (A) and Tgfbr2 (B) are shown. Scale bar shows 100 μm.

We also performed immunostaining to investigate TGF-beta receptor expression in kidney at corresponding time points. In PNW 3.5 wild-type mice, there was only faint staining for TGFβ Receptor 1, mainly in renal tubules. In *Pkd1*^*L*3/*L*3 ^kidney, TGFβ Receptor 1 was strongly expressed in cyst epithelium and interstitium (Figure 4A). The over-expression pattern was also seen with TGFβ Receptor 2. Significantly increased TGFβ Receptor 2 immunostaining was observed in *Pkd1*^*L*3/*L*3 ^kidney in cyst epithelium, interstitium and distorted glomeruli, in contrast to the weak staining in wild-type littermates (Figure 4B).

### Confirmation of PKD Custom Microarray Findings Through Quantitative Real-Time PCR Analyses

**Figure 5:**
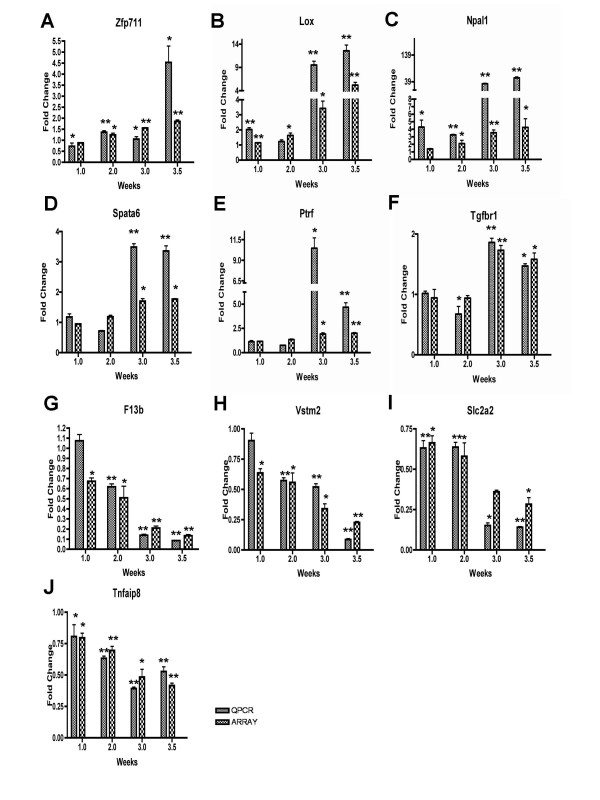
**RT-PCR analyses of selected genes (*Zfp711, Lox, Npal1, Spata6, Ptrf, Tgfbr1, F13b, Vstm2, Slc2a2 and Tnfaip8*) of *Pkd1*^*L*3/*L*3 ^and control mice at different stages**. RNA was extracted from *Pkd1*^*L*3/*L*3 ^mice and age-matched control littermates at PNW 1, 2, 3 and 3.5. Three micrograms of total RNA from each group were reverse transcribed and cDNA equivalent to 10 ng total RNA was used for the PCR reactions. The graphs represent the relative change compared to control groups after normalization against *Gapdh *expression (mean ± SD of triplicate PCR reactions of pooled RNA; *n *= 3; *, *P *< 0.05; **, *P *< 0.01 compared with wild-type mice, by Student's *t*-test).

To further confirm the differential gene expression at each time point observed with the PKD custom microarray, we validated their differential expression patterns using real time PCR analysis. We randomly selected six up-regulated genes, *Zfp711, Lox, Npal1, Spata6, Ptrf and Tgfβr *(Figure 5A–F), and four down-regulated genes, *F13b, Vstm2, Slc2a2 *and *Tnfaip8 *(Figure 5G–J), and verified their expression level by quantitative real time PCR, normalized against the housekeeping gene *Gapdh*. The expression pattern of all selected genes was similar to and consistent with the microarray results (Figure 5A–J). In general, PCR-based estimates of expression change were larger than those observed in the microarray study. These genes may prove useful as potential therapeutic target candidates.

## Conclusion

Comprehensive gene expression profiles from early to end stage ADPKD were generated from *Pkd1*^*L*3/*L*3 ^mice, an improved disease model. Several distinct early expression signatures of differentially expressed genes with potential roles in cystogenesis were identified. These genes are involved in several pathways including cell proliferation, apoptosis, transport, and immune defenses. A variety of biochemical pathways are clearly involved in the pathogenesis of ADPKD, both in initial cystogenesis and throughout disease progression, with extensive crosstalk between different pathways. Further investigation of these genes and their associated networks may provide insight into the possible pathogenic mechanisms of ADPKD development, and identify potential diagnostic and prognostic markers and novel therapeutic targets.

## Competing interests

The authors declare that they have no competing interests.

## Authors' contributions

WCC and HL were involved in designing the study. WCC and YST were involved in the animal breeding, tissue collection, RNA purification, microarray analysis, interpretation, and qPCR validation. WCC was involved in the statistical analysis and involved in data interpretation and writing of the manuscript.

## Supplementary Material

Additional file 1 **Materials and methods.** The animal experiment, microarray, data analysis and Real-Time PCR were described detail in this file. Click here for file

Additional file 2 **Postnatal growth retardation, kidney enlargement and progression of renal cystic lesions in *Pkd1*^*L*3/*L*3 ^mutant mice.** (A) Gross appearance of *Pkd1*^*L*3/*L*3 ^mutant mice and their normal littermates at postnatal weeks (PNW) 1, 2, 3, and 3.5. Note the progressive abdominal enlargement in *Pkd1*^*L*3/*L*3 ^mutant mice. (B) Kidney morphology of *Pkd1*^*L*3/*L*3 ^mutant mice and normal littermates at PNW 1, 2, 3, and 3.5. The enlarged pale and semi-translucent kidney in *Pkd1*^*L*3/*L*3 ^mutant mice was first seen at PNW 2 and became more evident at later time points. (C) H&E-stained kidney sections at PNW 1, 2, 3, and 3.5. Numerous cysts formed and progressively enlarged in kidney parenchyma in both cortex and medulla. (Scale bar = 2000 μm) All images (A-C) are representative of findings in at least three mice per genotype in two independent experiments. Click here for file

Additional file 3 **Early mortality, kidney enlargement, and loss of renal function in *Pkd1*^*L*3/*L*3 ^mutant mice.** Most mutant mice died before 28 days of age. Data from both males and females has been pooled. (A) Survival curves of wild-type (filled circle; *n *= 15), *Pkd1*^*L*3/+ ^(open circle; *n *= 15), and *Pkd1*^*L*3/*L*3 ^(filled triangle; *n *= 15) mice. Most mutant mice died before 28 days of age. Data from both males and females has been pooled. Body weight (B), ratio of kidney weight to body weight (%BW; C) and kidney volume (D) of wild-type, *Pkd1*^*L*3/+^, and *Pkd1*^*L*3/*L*3 ^mutant mice were recorded at PNW 1, 2, 3, and 3.5. (E) Deterioration in renal function in *Pkd1*^*L*3/*L*3 ^mutant mice. Mice were sacrificed and sera collected at PNW 1, 2, 3, and 3.5. BUN was determined from plasma concentration; *n *= 6 at each genotype and age; *, *P *< 0.05.; **, *P *< 0.01 compared with wild-type or heterozygous mice, by Student's *t*-test). Click here for file

Additional file 4 **Completed gene list of all transcripts significantly changed between wild-type and *Pkd1*^*L*3/*L*3 ^mice at one or more time points.** A table showing all transcripts identified as changing significantly between wild-type and *Pkd1*^*L*3/*L*3 ^mice (Raw: intensity of *Pkd1*^*L*3/*L*3 ^mice; Control: intensity of wild-type; *t*-test, *P *< 0.05; relative increase > 1.5 or < 1/1.5), and ranked by the ratio at PNW3.5. We also listed the functional group and clustering number in this table including relative change at each time point, accession number, gene symbol and the number of functional group and cluster group. Click here for file

Additional file 5 **Functional classification of genes differentially expressed between *Pkd1*^*L*3/*L*3 ^mice and age-matched control littermates.** Numbers of genes differentially expressed between *Pkd1*^*L*3/*L*3 ^mice and age-matched control littermates at each time point, grouped by functional categories. Click here for file

Additional file 6 **List of top 10 genes differentially expressed at each functional group.** The 10 genes with the largest changes in expression in wild-type and *Pkd1*^*L*3/*L*3 ^mice in each functional group, including relative change at each timepoint, accession number and gene symbol (Raw: intensity of *Pkd1*^*L*3/*L*3 ^mice; Control: intensity of wild-type). Click here for file

Additional file 7 **Primers and experimental conditions of *Gapdh *and validated genes confirmed with real-time RT-PCR.** This table contains a complete list and details of amplification conditions for the primers used in the RT-PCR analysis. Click here for file

Additional file 8 **Differentially expressed genes in the kidney of *Pkd1*^*L*3/*L*3 ^mice and age-matched control littermates at PNW 1of ADPKD.** List of genes that were differentially expressed in wild-type and *Pkd1*^*L*3/*L*3 ^mice at PNW 1. Click here for file

Additional file 9 **Top 5 networks generated from IPA at PNW 2, 3 and 3.5.** List of Ingenuity networks generated by the focus genes that were differentially expressed in wild-type and *Pkd1*^*L*3/*L*3 ^mice at PNW 2, 3 and 3.5. Click here for file
