# Supplementary material for: Gene expression in early and progression phases of autosomal dominant polycystic kidney disease
Source: BMC Res Notes. 2008 Dec 21;1:131. doi: 10.1186/1756-0500-1-131 (PMC2632667; doi:10.1186/1756-0500-1-131)
Supplement: Additional file 1 — Materials and methods. The animal experiment, microarray, data analysis and Real-Time PCR were described detail in this file. [file 1756-0500-1-131-S1.doc]

**Materials and Methods**

**Animals**

To obtain a C57BL/6 congenic strain, *Pkd1^L3/+^* mice were bred with C57BL/6 wild-type mice for over eight generations. *Pkd1^L3/L3^* ADPKD model mice and wild-type mice were bred by intercrossing *Pkd1^L3/+^* heterozygotes at the Animal Facility of the Institute of Molecular Biology at Academia Sinica, Taiwan. Use of animals was approved by the Institutional Animal Care and Use Committee in the Institute of Molecular Biology, Academia Sinica, Taiwan (protocol number MMiIMBLH2007075). All experiments conformed to the Animal Welfare Act Guide for Use and Care of Laboratory Animals, and the US Government Principles of the Utilization and Care of Vertebrate Animals Used in Testing, Research and Training guidelines on the ethical use of animals.

**Plasma Urea Determination**

Blood urea nitrogen (BUN) levels were measured from collected serum by the Taipei Institute of Pathology.

**Kidney Histology**

Dehydrated tissues were embedded in paraffin, and sectioned on a microtome at 5 μm for hematoxylin-eosin (H&E) staining. H&E stained sections were used to determine the cyst volume density by point counting stereology.

For immunostaining, a standard immunoperoxidase protocol (Vectastain ABC kit; Vector Laboratories) was used. After blocking with goat serum, sections were incubated with primary antibodies (anti-TGF beta receptor type 1 and 2; R&D Systems). Samples were then rinsed and incubated with streptavidin-conjugated peroxidase, using 3-amino-9-ethyl-carbazole or diaminobenzidine as a chromogen. Samples were also counterstained with H&E.

**cDNA Microarray**

DNA microarray analysis was performed by the Microarray Core Facility of the Institute of Molecular Biology, Academia Sinica, Taiwan (http://www.imb.sinica.edu.tw/mdarray/).

**Microarray Analysis**

A total of 13 microarray data sets were generated with RNA samples from age-matched control and disease littermates at the four time points, with at least three biological replicates for each time point (PNW1, *n =* 3; PNW2, *n =* 4; PNW3, *n =* 3; PNW3.5, *n =* 3). Expression data for all replicate microarray experiments were analyzed with GeneSpring 6.2 (Silicon Genetics). LOWESS normalization was used to normalize data obtained from GeneSpring. Condition Canonical pathway and gene network analyses used the Ingenuity Pathways Analysis software package V5.0 (Ingenuity Systems). The data discussed in this publication have been deposited in NCBI's Gene Expression Omnibus and are accessible through GEO Series accession number GSE13452 (http://www.ncbi.nlm.nih.gov/geo/query/acc.cgi?acc=GSE13452).

**Identifying Disease Progression Genes**

A two-step gene selection process was used to identify a subset of disease progress genes from microarray expression profiles. The control microarray data was first analyzed to find genes with a very low variation in expression across the four timepoints. Genes that satisfied this criterion were chosen for further analysis in the disease strain, and genes that showed increasing rates of differential expression in the control and disease model arrays (correlation coefficient > 0.7 and *P* < 0.05) were selected as candidate disease progress genes (5 genes, see Table 2). The correlations of each gene were separately computed by Pearson statistics (S-PLUS, Insightful Corporation). Positive correlation coefficients represented a increase in gene expression.

**Quantitative Real-Time PCR**

Total RNA was isolated from kidney tissue from *Pkd1^L3/L3^* and age-matched control littermates using TRIZOL reagent (Invitrogen) according to manufacturer’s instructions. Fluorescence detection temperatures for different PCR products are listed (see Additional file 1), with a subsequent melting curve analysis (continuous fluorescence detection from 65°C to 95°C with a temperature slope of 0.1°C/second). The expression of a target gene was normalized to the constitutively expressed *Gapdh* gene in the same sample.
