# Supplementary figures and images for: Gene expression in early and progression phases of autosomal dominant polycystic kidney disease
Source: BMC Res Notes. 2008 Dec 21;1:131. doi: 10.1186/1756-0500-1-131 (PMC2632667; doi:10.1186/1756-0500-1-131)

## Slide 1
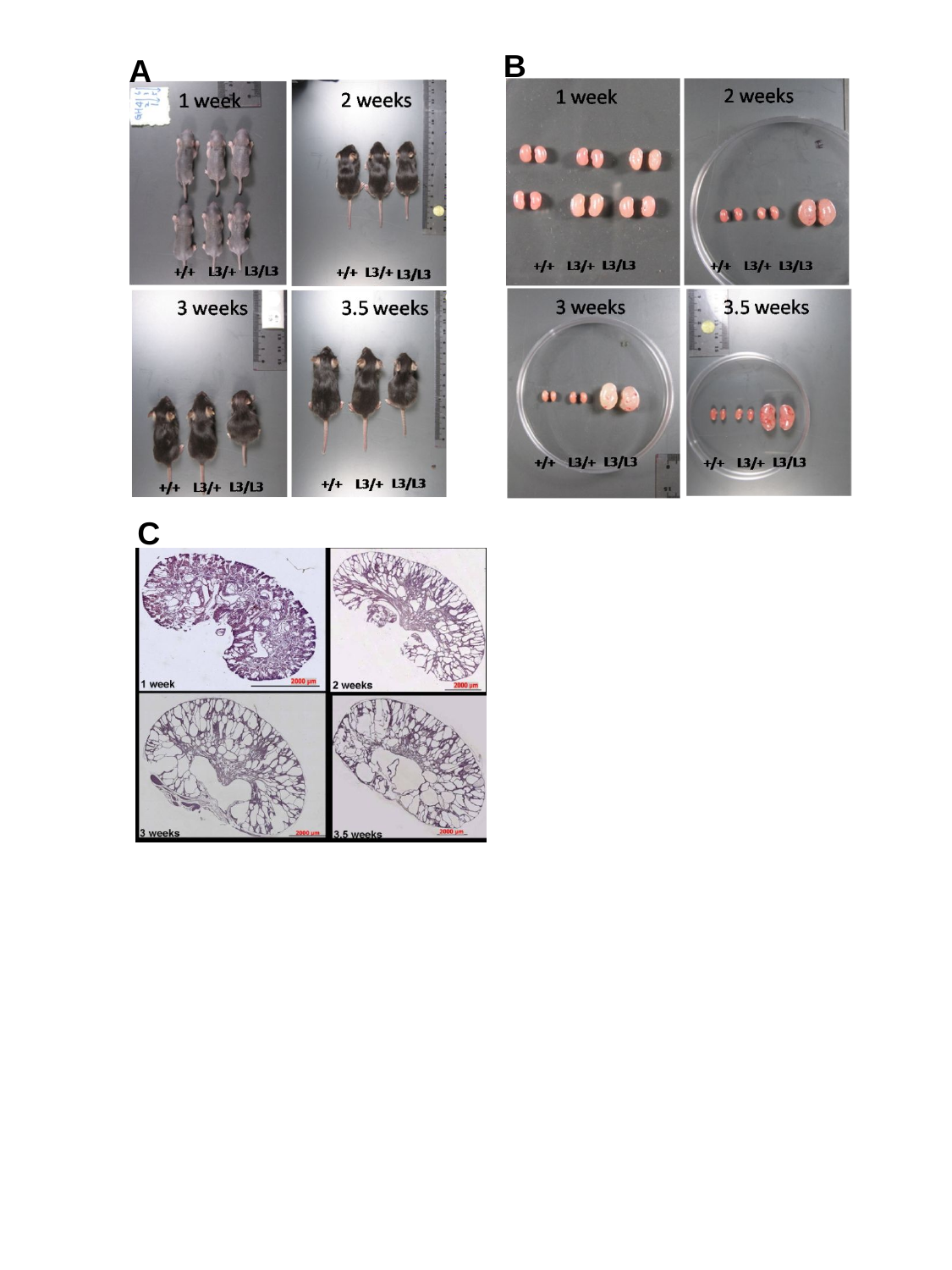

B
A
C

Supplement: Additional file 2 — Postnatal growth retardation, kidney enlargement and progression of renal cystic lesions in Pkd1L3/L3 mutant mice. (A) Gross appearance of Pkd1L3/L3 mutant mice and their normal littermates at postnatal weeks (PNW) 1, 2, 3, and 3.5. Note the progressive abdominal enlargement in Pkd1L3/L3 mutant mice. (B) Kidney morphology of Pkd1L3/L3 mutant mice and normal littermates at PNW 1, 2, 3, and 3.5. The enlarged pale and semi-translucent kidney in Pkd1L3/L3 mutant mice was first seen at PNW 2 and became more evident at later time points. (C) H&E-stained kidney sections at PNW 1, 2, 3, and 3.5. Numerous cysts formed and progressively enlarged in kidney parenchyma in both cortex and medulla. (Scale bar = 2000 μm) All images (A-C) are representative of findings in at least three mice per genotype in two independent experiments. [file 1756-0500-1-131-S2.ppt]

## Slide 1
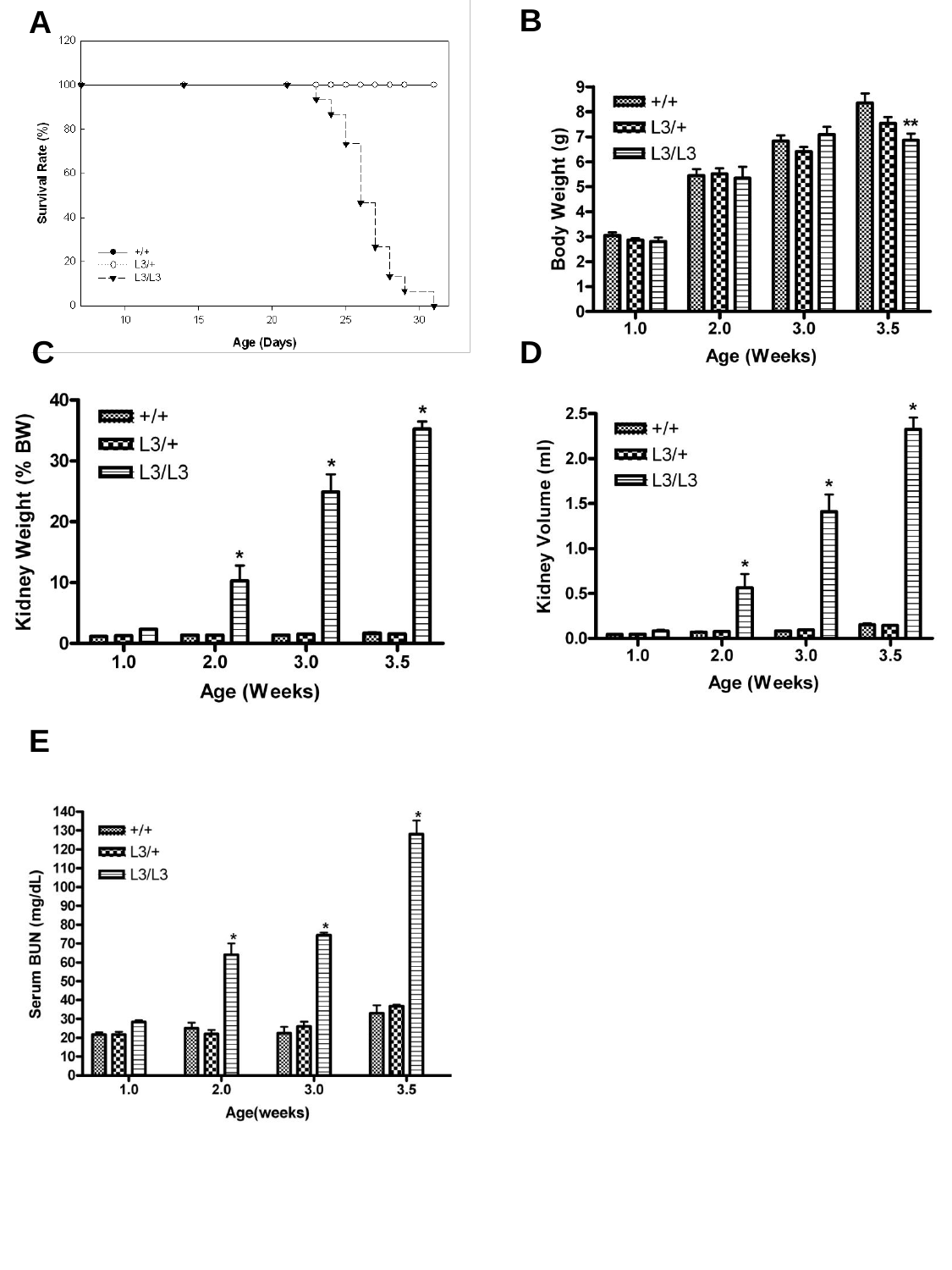

B
A
C
D
E

Supplement: Additional file 3 — Early mortality, kidney enlargement, and loss of renal function in Pkd1L3/L3 mutant mice. Most mutant mice died before 28 days of age. Data from both males and females has been pooled. (A) Survival curves of wild-type (filled circle; n = 15), Pkd1L3/+ (open circle; n = 15), and Pkd1L3/L3 (filled triangle; n = 15) mice. Most mutant mice died before 28 days of age. Data from both males and females has been pooled. Body weight (B), ratio of kidney weight to body weight (%BW; C) and kidney volume (D) of wild-type, Pkd1L3/+, and Pkd1L3/L3 mutant mice were recorded at PNW 1, 2, 3, and 3.5. (E) Deterioration in renal function in Pkd1L3/L3 mutant mice. Mice were sacrificed and sera collected at PNW 1, 2, 3, and 3.5. BUN was determined from plasma concentration; n = 6 at each genotype and age; *, P < 0.05.; **, P < 0.01 compared with wild-type or heterozygous mice, by Student's t-test). [file 1756-0500-1-131-S3.ppt]

## Slide 1
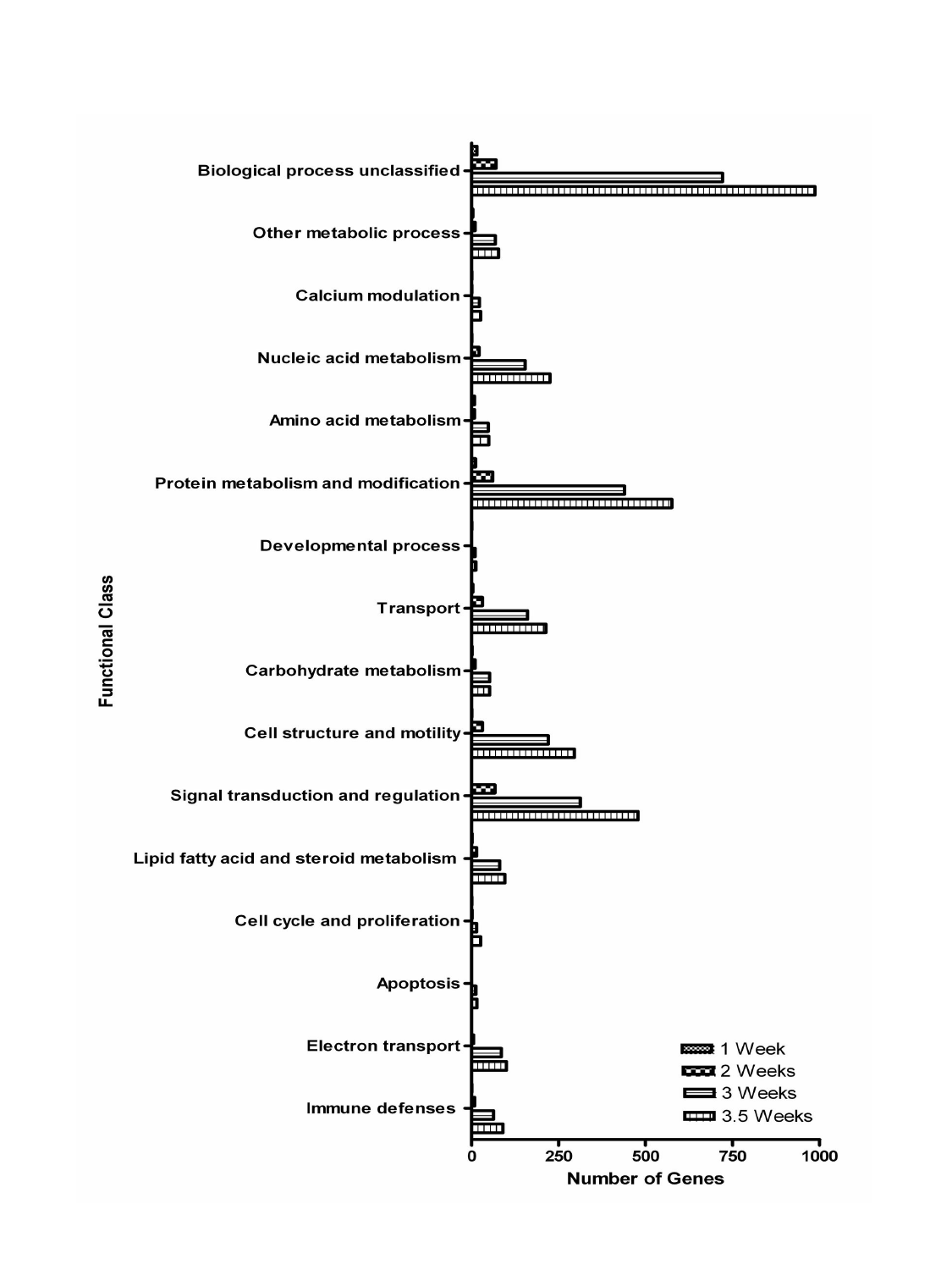

Supplement: Additional file 5 — Functional classification of genes differentially expressed between Pkd1L3/L3 mice and age-matched control littermates. Numbers of genes differentially expressed between Pkd1L3/L3 mice and age-matched control littermates at each time point, grouped by functional categories. [file 1756-0500-1-131-S5.ppt]
