# Supplementary material for: Gene expression in early and progression phases of autosomal dominant polycystic kidney disease
Source: BMC Res Notes. 2008 Dec 21;1:131. doi: 10.1186/1756-0500-1-131 (PMC2632667; doi:10.1186/1756-0500-1-131)
Supplement: Additional file 7 — Primers and experimental conditions of Gapdh and validated genes confirmed with real-time RT-PCR. This table contains a complete list and details of amplification conditions for the primers used in the RT-PCR analysis. [file 1756-0500-1-131-S7.doc]

**Table S3. Primers and experimental conditions of Gapdh and validated genes confirmed with real-time RT-PCR.**

| **Symbol** | **Primer Pair** | **Annealing  Temp (°C)** | **Elongation**  **Time (s)** |
| --- | --- | --- | --- |
| *Zfp711* | (F) 5'-GTGGTGGTGTTCCTGGTTCTACTG-3' | 52 | 6 |
|  | (R) 5'-TTTTCAATGGCGTGTTCCCC-3' |  |  |
| *Lox* | (F) 5'-CTACGATTTCCGCAAAGAGTGAAG-3' | 55 | 6 |
|  | (R) 5'-CATCAAGCAGGTCATAGTGGCTG-3' |  |  |
| *Npal1* | (F) 5'-TTGGGGTCAACTGTGATGGTTATC-3' | 54 | 10 |
|  | (R) 5'-CAGAAGAGACGGAAAACGCTCC-3' |  |  |
| *Spata6* | (F) 5'-TGGCAGAGACTGTGAACGAGATG-3' | 55 | 10 |
|  | (R) 5'-GCTTTGGTGTTGTTGAAGGTGC-3' |  |  |
| *Ptrf* | (F) 5'-GGAGGGGATGAAAACCACAAAC-3' | 55 | 10 |
|  | (R) 5'-CACCAGAACACAGCAACTCAAGG-3' |  |  |
| *F13b* | (F) 5'-TGAGGAAAAGGAGAAGGTAGCCTG-3' | 56 | 10 |
|  | (R) 5'-ACTTGCCAGTAACCCATCAACAAC-3' |  |  |
| *Vstm2* | (F) 5'-CAACTCTGCCAACCAACGAATG-3' | 55 | 10 |
|  | (R) 5'-GCTGTCCGCTCCGTAGATTTTAC-3' |  |  |
| *Slc2a2* | (F) 5'-TGTCCAGAAAGCCCCAGATACC-3' | 55 | 10 |
|  | (R) 5'-TCCCATTGATTCCTGAGAACTGC-3' |  |  |
| *Tnfaip8* | (F) 5'-GGGCAAAATGGTATCCAAATCC-3' | 56 | 6 |
|  | (R) 5'-TTGATGACCGTCTTGATGAGGTTC-3' |  |  |
| *Wnt7a* | (F) 5'- GCCTTCACCTATGCGATTATC-3' | 58 | 16 |
|  | (R) 5'- CCTCGTTGTATTTGTCCTTGAG-3' |  |  |
| *Slc9a3r1* | (F) 5'-TGGAGAAGGAGACGCATCAG -3' | 59 | 11 |
|  | (R) 5'- TGAAGCCATAGCCATTGGG-3' |  |  |
| *Ppap2b* | (F) 5'- AAACGATGCTGTGCTCTGTGCG-3' | 57 | 8 |
|  | (R) 5'- TTTGGCGATGTCGGTGAAGG-3' |  |  |
| *Wnt8b* | (F) 5'- TCCGAGGAGAATGCTTCCCATC-3' | 56 | 6 |
|  | (R) 5'- ATTGTTCACTGACCAGGCGTGG-3' |  |  |
| *Wisp3* | (F) 5'- ACTTGTCCTCCTGGGGTGAGTTTG-3' | 58 | 10 |
|  | (R) 5'- GAACAGTGGGTGGGGCTGAAATAC-3' |  |  |
| *Tax1bp3* | (F) 5'-CTCGGAAGATAAAACAGACAAGGG -3' | 57 | 10 |
|  | (R) 5'-TAGACGGTGGCTGACTGCTAAGAC -3' |  |  |
| *Frat2* | (F) 5'-ATGCCTTGCCGGAGAGAGGAG-3' | 55 | 12 |
|  | (R) 5'-GGTCTCTGCTGACCCCGTCGG-3' |  |  |
| *Tgfbr1* | (F) 5'-CTGCCATAACCGCACTGTCATTC-3' | 55 | 9 |
|  | (R) 5'-GCCAAACTTCTCCAAACCGACC-3' |  |  |
| *Dkk1* | (F) 5'-TGTTTGCGTCCTTCGGAGATG-3' | 58 | 11 |
|  | (R) 5'-GGTAGGGCTGGTAGTTGTCAAGAG-3' |  |  |
| *Dkk3* | (F) 5'-CCCAACTATCACAATGAGACCAGC-3' | 53 | 5 |
|  | (R) 5'-TCAGAAAAGACCACCTGTCCACTC-3' |  |  |
| *Fzd5* | (F) 5'-TCCCTCTCTTCTGCTGCTGTTG-3' | 59 | 11 |
|  | (R) 5'-GGCTTGTGGTAGTCAGGCAAAC-3' |  |  |
| *Gm784* | (F) 5'-CAGTGCCAGTCATCAATGGAGAG-3' | 55 | 11 |
|  | (R) 5'-TGCCAGTTGGTGCTATCAGAGG-3' |  |  |
| *Lrp6* | (F) 5'-TGTTTCTGGATTATTGTCCCCG-3' | 53 | 9 |
|  | (R) 5'-CGTTCTATCTTTGGCACTTCTCCC-3' |  |  |
| *Wnt2b* | (F) 5'-ACTCCCCTGACTACTGTGTCTTGG-3' | 57 | 10 |
|  | (R) 5'-TCCAGCCACTCTGCCTTCTTAG-3' |  |  |
| *Wnt7b* | (F) 5'-CTGCCTTCACCTATGCCATCAC-3' | 57 | 5 |
|  | (R) 5'-TGTAGTAGCCTTGCTTCTCCCG-3' |  |  |
| *Wisp1* | (F) 5'-GGACATCCAACTACACATCAAGGC-3' | 57 | 12 |
|  | (R) 5'-GGGTAAGATTCCAAGTCAGCAAAG-3' |  |  |
| *Tle6* | (F) 5'-ACTTCCCACAGACAGAGCAGTGAC-3' | 54 | 5 |
|  | (R) 5'-ACCATCATTTCAGAGACGACGC-3' |  |  |
| *Sostdc1* | (F) 5'-CCATTCATCTCTCTCTCATTCCCC-3' | 56 | 12 |
|  | (R) 5'-ATTTGGTGGACCGCAGTTCC-3' |  |  |
| *Ncstn* | (F) 5'-CAGGATTGTGTGGGGGAAACTC-3' | 56 | 12 |
|  | (R) 5'-GGCAAGACCAGCGATTCTACTG-3' |  |  |
| *Bmp3* | (F) 5'-TTGATGAGCAGACCCTGAAGAAG-3' | 55 | 9 |
|  | (R) 5'-GGTGGCGTGATTTGATGGTTTC-3' |  |  |
| *Gdf10* | (F) 5'-TCAGGTGCTGGACTTTGACGAG-3' | 58 | 9 |
|  | (R) 5'-TGGATGGGCGGACAATCTTG-3' |  |  |
| *Klk1b26* | (F)  5'- TTTGTGGGGGTGTCCTGTTG -3 | 55 | 12 |
|  | (R) 5'- TTGCTGACCAATCGGTGCTGAG -3' |  |  |
| *Klk1b8* | (F) 5'- GGTGGCTGTGTATGACAACAAGG -3' | 55 | 12 |
|  | (R) 5'- GCTTTTGCTGACCAATCGGTG -3' |  |  |
| *Klk1b5* | (F) 5'- TCTCCAGTGTGTGAACCTCAAGC -3' | 56 | 12 |
|  | (R) 5'- TCGCCATAGTTTCTCTTATCCAGG -3' |  |  |
| *Klk3* | (F) 5'- CCAGCATAAAGTCAGCGGACAAC -3' | 55 | 12 |
|  | (R) 5'- TGCGACACACAAAGGCATCG -3' |  |  |
| *Ptrf* | (F) 5'-GGAGGGGATGAAAACCACAAAC-3' | 55 | 12 |
|  | (R) 5'-CACCAGAACACAGCAACTCAAGG-3' |  |  |
| *F13b* | (F) 5'-TGAGGAAAAGGAGAAGGTAGCCTG-3' | 56 | 12 |
|  | (R) 5'-ACTTGCCAGTAACCCATCAACAAC-3' |  |  |
| *GAPDH* | (F) 5'-CCTGGAGAAACCTGCCAAGTATG-3' | 55 | 12 |
|  | (R) 5'-GTGGGAGTTGCTGTTGAAGTCG-3' |  |  |
