# Supplementary material for: Gene expression in early and progression phases of autosomal dominant polycystic kidney disease
Source: BMC Res Notes. 2008 Dec 21;1:131. doi: 10.1186/1756-0500-1-131 (PMC2632667; doi:10.1186/1756-0500-1-131)
Supplement: Additional file 8 — Differentially expressed genes in the kidney of Pkd1L3/L3 mice and age-matched control littermates at PNW 1of ADPKD. List of genes that were differentially expressed in wild-type and Pkd1L3/L3 mice at PNW 1. [file 1756-0500-1-131-S8.doc]

**Table S4. Differentially expressed genes in the kidney of *Pkd1^L3/L3^* mice and age-matched control littermates at PNW 1of ADPKD.**

| **Gene Symbol** | **Description** | **Relative Change** | ***P***-value | **Category** |
| --- | --- | --- | --- | --- |
| *Il17rb* | Interleukin 17 receptor B | 1.82 | 4.08E-02 | immune defenses |
| *Idh1* | Isocitrate dehydrogenase 1, soluble | 0.66 | 1.52E-02 | electron transport |
| *Zfp106* | Zinc finger protein 106 homolog | 1.65 | 1.02E-02 | electron transport |
| *Aldh1a1* | Aldehyde dehydrogenase 1 family, member A1 | 2.05 | 1.14E-02 | apoptosis |
| *Ngfg* | Nerve growth factor, gamma subunit | 0.52 | 1.23E-03 | apoptosis |
| *Gas2l1* | Growth arrest-specific 2 like 1 | 1.51 | 4.35E-02 | cell cycle and proliferation |
| *Gm784* | Low density lipoprotein-related protein 1 | 0.66 | 6.38E-03 | lipid, fatty acid, and steroid metabolism |
| *Aadac* | Arylacetamide deacetylase | 0.66 | 1.61E-02 | lipid, fatty acid, and steroid metabolism |
| *Akr1c2* | Aldo-keto reductase family 1, member C2 | 0.66 | 2.33E-02 | lipid, fatty acid, and steroid metabolism |
| *Krt18* | Keratin 18 | 1.52 | 2.99E-02 | cell structure and motility |
| *Car15* | Carbonic anhydrase 15 | 0.61 | 4.95E-02 | carbohydrate metabolism |
| *Slc2a2* | Solute carrier family 2, member 2 | 0.66 | 2.57E-02 | carbohydrate metabolism |
| *Slc5a12* | Solute carrier family 5, member 12 | 0.66 | 2.44E-02 | transport |
| *Fxyd2* | FXYD domain containing ion transport regulator 2 | 0.65 | 9.08E-03 | transport |
| *Abcg2* | ATP-binding cassette, sub-family G, member 2 | 0.63 | 6.28E-04 | transport |
| *Klk1b22* | Kallikrein 1-related peptidase b22 | 0.65 | 1.25E-02 | Protein metabolism and modification |
| *Klk1b9* | Kallikrein 1-related peptidase b9 | 0.58 | 1.60E-02 | Protein metabolism and modification |
| 4931432M23Rik | Plasma kallikrein-like protein 4 | 1.61 | 4.90E-02 | Protein metabolism and modification |
| *Lrrc8e* | Leucine rich repeat containing 8 family, member E | 1.52 | 7.92E-04 | Protein metabolism and modification |
| *Slitrk6* | SLIT and NTRK-like family, member 6 | 0.66 | 3.12E-02 | Protein metabolism and modification |
| *Klk1b11* | Kallikrein 1-related peptidase b11 | 0.63 | 4.39E-04 | Protein metabolism and modification |
| *Gfer* | Growth factor, augmenter of liver regeneration | 0.34 | 3.50E-02 | Protein metabolism and modification |
| *Klk3* | Kallikrein-related peptidase 3 | 0.44 | 3.24E-03 | Protein metabolism and modification |
| *Klk1* | Kallikrein 1 | 0.50 | 9.23E-03 | Protein metabolism and modification |
| *Klk1b5* | Kallikrein 1-related peptidase b5 | 0.48 | 3.34E-03 | Protein metabolism and modification |
| *Ctsl2* | Cathepsin L2 | 0.64 | 4.90E-02 | Protein metabolism and modification |
| *Vwf* | Von Willebrand factor | 1.59 | 2.50E-02 | Protein metabolism and modification |
| 1700127d06rik | RIKEN cDNA 1700127D06 gene | 0.50 | 4.90E-03 | Protein metabolism and modification |
| *Tmem27* | Transmembrane protein 27 | 0.65 | 4.19E-02 | Protein metabolism and modification |
| *Klk1b8* | Kallikrein 1-related peptidase b8 | 0.48 | 4.35E-04 | amino acid metabolism |
| *Klk1b26* | Kallikrein 1-related peptidase b26 | 0.49 | 4.85E-03 | amino acid metabolism |
| *Pah* | Phenylalanine hydroxylase | 0.58 | 6.10E-03 | amino acid metabolism |
| *Gatm* | Glycine amidinotransferase | 0.64 | 6.99E-03 | amino acid metabolism |
| *Zfp40* | Zinc finger protein 40 | 0.59 | 2.91E-02 | nucleic acid metabolism |
| *Pol* | Retrovirus-related Pol polyprotein | 1.58 | 4.61E-02 | nucleic acid metabolism |
| 4930504H06Rik | RIKEN cDNA 4930504H06 gene | 0.66 | 1.45E-03 | calcium modulation |
| *Acss1* | Acetyl-CoA synthetase short-chain family member 1 | 0.64 | 1.59E-03 | Other metabolism |
| X05165 | X05165 | 1.62 | 2.48E-02 | biological process unclassified |
| X05165 | X05165 | 1.56 | 2.81E-02 | biological process unclassified |
| *Vstm2* | V-set and transmembrane domain containing 2 | 0.63 | 1.27E-02 | biological process unclassified |
| ENSMUSG00000046208 |  | 1.74 | 4.24E-02 | biological process unclassified |
| *Gldn* | Gliomedin | 1.68 | 2.30E-03 | biological process unclassified |
| ENSMUSG00000043379 |  | 1.52 | 1.36E-02 | biological process unclassified |
| *Qki* | Quaking homolog, KH domain RNA binding | 0.57 | 3.01E-02 | biological process unclassified |
| ENSMUSG00000036552 |  | 1.58 | 4.29E-02 | biological process unclassified |
| 1700010D01Rik | RIKEN cDNA 1700010D01 gene | 0.59 | 4.80E-03 | biological process unclassified |
| *Armc4* | Armadillo repeat containing 4 | 0.64 | 9.26E-03 | biological process unclassified |
| ENSMUSG00000006183 |  | 1.52 | 7.04E-03 | biological process unclassified |
| D730048I06Rik | RIKEN cDNA D730048I06 gene | 0.66 | 7.71E-03 | biological process unclassified |
| *F13b* | Coagulation factor XIII, B polypeptide | 0.67 | 1.51E-02 | biological process unclassified |
