# Supplementary material for: Gene expression in early and progression phases of autosomal dominant polycystic kidney disease
Source: BMC Res Notes. 2008 Dec 21;1:131. doi: 10.1186/1756-0500-1-131 (PMC2632667; doi:10.1186/1756-0500-1-131)
Supplement: Additional file 9 — Top 5 networks generated from IPA at PNW 2, 3 and 3.5. List of Ingenuity networks generated by the focus genes that were differentially expressed in wild-type and Pkd1L3/L3 mice at PNW 2, 3 and 3.5. [file 1756-0500-1-131-S9.doc]

**Table S5-1. Top 5 networks generated from IPA for differentially expressed genes in the Pkd1L3/L3 mice at PNW 2.** a

| **Network ID** | **Genes in network** | **Score** | **# No. focus**  **genes** | **Top categories** |
| --- | --- | --- | --- | --- |
| 1 | **↓COL4A3, ↑CTGF, ↑ELF5, ↑F2, ↑FANCA, ↓GCNT1, ↓GFER, ↓GHR, ↑HMOX1, ↑KRT8, ↑LGALS3, ↑LOX, ↑LRRN3, ↓NOX4, ↓NPHS2, ↑PMAIP1, ↑SCNN1B, ↑SCNN1G, ↑STAT5B, ↑STYK1, ↑TAGLN, ↑TGFB3, ↑TNC, ↓VCAM1, ↓WNK1** | 49 | 25 | Genetic Disorder, Metabolic Disease, Tissue Morphology |
| 2 | **↓ABCG2, ↑AQP3, ↑COL12A1, ↓CRYZ,↓CYP2J5, ↓FMO2, ↑HSD11B2, ↓KLK3, ↓KLK1(includes EG:3816), ↓KLK1B9, (↑B3GN-T6)** | 27 | 16 | Cancer, Cellular Growth and Proliferation, Drug Metabolism |
| 3 | **↑FXYD4, ↑KIF23, ↑LOXL1, ↓LRP2, ↓MBNL2, ↑NLGN3, ↑OAT, ↑PCP2, ↑POSTN, ↓SOST, ↑TMSB10, ↓TTR, ↓WNK1** | 27 | 16 | Cell Death, Nervous System Development and Function, Cardiovascular System Development and Function |
| 4 | **↓ACSS1, ↓ATF7IP2(includes EG:80063), ↑CPLX1, ↓DIDO1, ↑FAM129A, ↓GATM, ↑HSD11B2, ↓KLK1 (includes EG:16612), ↓PTPRO, ↑REPS1, ↑SCEL, ↑SLC27A5, ↑SLC27A6, ↑TNC** | 23 | 14 | Cell Death, Connective Tissue Disorders, Cancer |
| 5 | **↑ACTG2, ↑AVPR2, ↑DAB1, ↓FBP1, ↓KL, ↑PENK, ↑RASGRF2, ↓S100G, ↓SLC13A1, ↓SMPDL3A, ↑STARD13, ↑TPM1, ↑TUBB2A,↑ZBTB16** | 23 | 14 | Cellular Assembly and Organization, Cardiovascular Disease, Cell Morphology |

*^a^*Genes whose expression was either increased or decreased in *Pkd1^L3/L3^* mice as a function of age were identified by correlation analysis across four time points: 1,2, 3 and 3.5 weeks.

**Table S5-2. Top 5 networks generated from IPA for differentially expressed genes in the Pkd1^L3/L3^ mice at PNW 3. ^a^**

| Network ID | Genes in network | Score | # No. focus  genes | Top Functions |
| --- | --- | --- | --- | --- |
| 1 | **↓ARL6IP1, ↑BBC3, ↑CALD1, ↑CCL6, ↑COL5A1, ↑COL5A3, ↓CPT2, ↓CPT1A, ↓CPT1B, ↓FBLN5, ↑GJA1, ↓HSPH1, ↓IGLL1, ↓LAMP2, ↑LOXL1, ↓LY6A, ↑MAP4, ↑MARCKSL1, ↑MYC, ↓NDRG1, ↑NPM1 (includes EG:18148), ↑PLS3, ↑RBP1, ↓RDH16, ↓RSAD2, ↓SARDH, ↓SCPEP1, ↑SERINC3, ↓SLC25A5, ↓SUCLA2, ↓VPREB1 (includes EG:7441), ↑ZAK, ↑ZBTB16** | 46 | 33 | Cancer, Cell Cycle, Hematological System Development and Function |
| 2 | **↓AIFM1, ↑ANXA5, ↑BIRC3, ↑C14ORF106,↑CDKN1A, ↑CENPF, ↓CFLAR, ↑CP, ↓CYCS, ↓DAPK1, ↑DTL, ↓FDX1, ↑FGF13, ↓GAS2, ↑GSN, ↓HBXIP, ↑KLF10, ↑LGALS3, ↓LOC374569, ↑NOC2L, ↑NUAK1, ↑PMAIP1, ↓PROC, ↑PROS1, ↑RPA3, ↑SART2, ↓SLC25A4, ↑SMC2, ↓SP100, ↓TCN2, ↓TNFSF10, ↑TNFSF15, ↑WISP1** | 46 | 33 | Cell Death, Cancer, Gastrointestinal Disease |
| 3 | **↓ACAT1, ↓ACY1, ↓ALDOB, ↓APOA2, ↑CCR1, ↑CD9, ↓CD63, ↑CRIP1, ↓DHCR24, ↑DSC2, ↓EPPK1, ↑FGG, ↓GK, ↓GPAM, ↑GPR34, ↓HMGCL, ↓HNF4A, ↓IDH3A, ↑ITGAM, ↑LOX, ↓PCK1, ↑PIK3AP1, ↓PPARGC1A, ↓PPARGC1B, ↓PRKAA2, ↑RBP4, ↑SCNN1B, ↑SCNN1G, ↓STX3, ↑STYK1, ↓WNK1** | 41 | 31 | Lipid Metabolism, Molecular Transport, Small Molecule Biochemistry |
| 4 | **↓ANK3, ↓ASS1, ↑CAMK2B, ↑CD44, ↑CDH1, ↓DPP4,↑FGF18, ↑FGFR2, ↓IRX1 (includes EG:79192), ↑ITGA11, ↓ITGAE, ↓KL, ↓LMO7, ↓LPL, ↓LRP2, ↓LRPAP1, ↑NES,↑PTPRC, ↓PTPRD, ↑PTPRS (includes EG:5802), ↑SERPINE2, ↓SLC9A3, ↑ST6GAL1,↓TLN2, ↑TOP2A, ↓TSPAN3, ↑VCL, ↓VLDLR, ↑ZNF202** | 37 | 29 | Cellular Growth and Proliferation, Embryonic Development, Developmental Disorder |
| 5 | **↓ACE2, ↓AGT, ↓AGTRL1, ↑APLN, ↑BOK, ↑CD47, ↓CEP350, ↓COL4A3, ↓COX5A, ↓COX5B, ↓COX6C (includes EG:1345), ↓CYP2C44, ↓CYP2D26, ↓CYP2J5, ↓FRAP1, ↑HOXA11, ↓HOXA9 (includes EG:3205), ↑HOXB2, ↑HOXB7, ↑HPR, ↑MYH10, ↓NOX4, ↓NR1H3, ↓OSBPL8, ↓PIM3,↓PPP2R3A (includes EG:5523), ↓REN, ↑SERINC2, ↓SLC4A1** | 37 | 29 | Cardiovascular System Development and Function, Organ Morphology, Cardiac Necrosis/Cell Death |

*^a^*Genes whose expression was either increased or decreased in *Pkd1^L3/L3^* mice as a function of age were identified by correlation analysis across four time points: 1,2, 3 and 3.5 weeks.

**Table S5-3. Top 5 networks generated from IPA for differentially expressed genes in the Pkd1^L3/L3^ mice at P3.5. ^a^**

| Network ID | Genes in network | Score | # No. focus  genes | Top Functions |
| --- | --- | --- | --- | --- |
| 1 | **↓ARL6IP1, ↑BBC3, ↑CALD1, ↑CCL6, ↑COL5A1, ↑COL5A3, ↓CPT2, ↓CPT1B, ↑CPT1C, ↑FAM129A, ↓FBLN5, ↑HIF3A, ↓HSPH1, ↑HTATIP2, ↓IGLL1, ↓LAMP2, ↑LOXL1, ↑MAP4, ↑MYC, ↑NPM1 (includes EG:18148), ↓PGK1, ↑PLS3, ↑RBP1, ↓RDH16, ↓RSAD2, ↓SARDH, ↓SCPEP1, ↓SEPT6, ↑SERINC3, ↓SLC25A5, ↓SUCLA2, ↓TP53BP2, ↓VPREB1 (includes EG:7441), ↑ZAK** | 45 | 34 | Hematological System Development and Function, Immune and Lymphatic System Development and Function, Tissue Morphology |
| 2 | **↓ACAT1, ↓ACY1, ↑COL3A1, ↑CRIP1, ↓CSE1L, ↓CYP2C44, ↓CYP2D26, ↓CYP2J5, ↓DAPK1, ↑DSC2, ↓EPPK1, ↑ESRRG, ↑FN1, ↑GABRE, ↓GK, ↓HMGCL, ↓HNF4A, ↓IDH3A, ↓IDH3B, ↓IDH3G, ↓KLK1B21, ↑LOX, ↑LPIN1, ↓NOX4, ↑NPNT, ↓NR1H3, ↓PCK1,↓PPARGC1A, ↓REN, ↓SCD, ↑SRCAP, ↑TGFB3** | 40 | 32 | Gene Expression, Lipid Metabolism, Small Molecule Biochemistry |
| 3 | **↓AKR1B1, ↓BCAN, ↑CNKSR3, ↑CTGF, ↑CXCL14, ↑DLL1, ↓ENPP2, ↑FBN1, ↑FGA, ↑FGF2, ↑FGG,↓GAL3ST1, ↓GLUL, ↑HSPG2 (includes EG:3339),↓IGFBP4, ↓IGFBP5, ↑ITGAM, ↓KNG1 (includes EG:3827), ↑LAMA5, ↑LCN2, ↓LRP2,↑MMP3, ↑MMP14, ↑NKD2, ↑NR4A2, ↓PI3, ↑SERPINA1,↑TGFA, ↑TIMP1, ↑TMSB10, ↓TTR** | 38 | 31 | Cancer, Cellular Movement, Cardiovascular System Development and Function |
| 4 | **↑ARHGAP11A, ↓C14ORF156, ↑CBX6, ↑CDH1, ↑CORO2A, ↓CRSP8, ↓DIO1,↓FABP2, ↑GNG10, ↑GPR56, ↑HDAC4, ↓HOXA9 (includes EG:3205), ↑HOXD3, ↓LMO7, ↑MKL1, ↓OSBPL8, ↑PBX3, ↑PBXIP1, ↓POLR2B, ↓POLR2K, ↑POLR3B, ↑POLR3H, ↑PRIM2A, ↓PTPRU, ↑RNMT, ↑SERINC2, ↑TAGLN, ↑TFF1, ↓THAP7, ↑WWC1, ↑ZFP36** | 38 | 31 | Cancer, Hematological Disease, Cellular Movement |
| 5 | **↓ADK, ↓AIFM1, ↑B4GALT1, ↑BAK1, ↑BIRC3, ↑C1S, ↑CKAP4, ↓CYCS, ↑DCAMKL1, ↓DNAJC13, ↑ECE1, ↓ELL2, ↑F2, ↓FDX1,↓GSTA4, ↓GSTO2, ↓HBXIP,↑IRF5, ↑LOXL2, ↓NCOA7, ↓PBEF1, ↑PLEK, ↑PMAIP1, ↓PPIF, ↑SERPINB6, ↑SERPINE2, ↑SERPING1, ↓SLC25A4, ↑UCK2 (includes EG:7371), ↓VLDLR** | 36 | 30 | Cell Morphology, Cellular Assembly and Organization, Cellular Compromise |

*^a^*Genes whose expression was either increased or decreased in *Pkd1^L3/L3^* mice as a function of age were identified by correlation analysis across four time points: 1,2, 3 and 3.5 weeks.
